# Diversity of Cultivable Microbes From Soil of the Fildes Peninsula, Antarctica, and Their Potential Application

**DOI:** 10.3389/fmicb.2020.570836

**Published:** 2020-09-03

**Authors:** Bailin Cong, Xiaofei Yin, Aifang Deng, Jihong Shen, Yongqi Tian, Shaoyun Wang, Huanghao Yang

**Affiliations:** ^1^The First Institute of Oceanography, Ministry of Natural Resources, Qingdao, China; ^2^College of Biological Science and Engineering, Fuzhou University, Fuzhou, China

**Keywords:** Antarctica, cultivable microbes, novel species, enzymes, antifungal natural product

## Abstract

To explore the diversity and application potential of Antarctic microorganisms, 1208 strains bacteria and fungi were isolated from 5 samples collected from the Fildes Peninsula during China’s 27th and 31st Antarctic expeditions. By using 16S and ITS sequence similarity alignment, 83 strains bacteria belonging to 20 genera and 30 strains fungi belonging to 7 genera were identified. Among them, 1 strains bacteria and 6 strains fungi showed low sequence similarity to the database, suggesting that they might be novel species. Physiological-biochemical characteristics showed that the identified bacteria could utilize many kinds of carbohydrates and that the identified fungi could produce several kinds of extracellular enzymes. The fungal strain MS-19, identified as *Aspergillus sydowii*, possesses the potential to produce antifungal activity agents based on an activity-guided approach. Further isolation yielded four polyketones: versicone A (**1**), versicone B (**2**), 4-methyl-5,6-dihydro-2H-pyran-2-one (**3**), and (*R*)-(+)-sydowic acid (**4**). It should be noted that **1** displayed strong activity against *Candida albicans*, with an MIC value of 3.91 μg/mL.

## Introduction

Antarctica is located at the southernmost point of the earth, and the climate of Antarctica is very different from that of other places due to the Antarctic circulation. Antarctica has a simple and weak ecosystem because of the cold, dry climate and low level of nutrition (Chown et al., 2015). Few animals and plants can survive in this cold environment. Because of the special habitat, microbes in Antarctica evolved extraordinary resistance to low temperature, hypersalinity and radiation (Dong et al., 2015).

Scientists have collected and analyzed many samples from Antarctica to explore the biological diversity of this continent. Because of the rigorous environment, flowering plants and vertebrates have been rarely observed, and for those that have been observed, their main habitat was near the ocean. Lichens, mosses, nematodes, tardigrades, springtails and mites are much more abundant in Antarctica compared with higher plants and animals (Velascocastrillon et al., 2014; Godinho et al., 2015). On the other hand, the microbes displayed extraordinary diversity. Cowan et al. reported the abundance of microbes discovered from hyperarid McMurdo Dry Valleys, and the dominant bacteria were *Acidobacteria*, *Actinobacteria*, and *Bacteroidetes* (Cowan et al., 2010). Teixeira et al. researched the bacterial diversity in rhizosphere soil from Antarctic vascular plants of Admiralty Bay and found that the most abundant phylum was *Firmicutes*, while *Bifidobacterium*, *Arcobacter* and *Faecalibacterium* were also prominent (Teixeira et al., 2010). In other warmer and wetter parts of Antarctica, such as the ice-free area of the Keller Peninsula, *Proteobacteria* are very abundant (Freckman and Virginia, 1997). Recently, research based on DNA sequencing was proven to be powerful for investigating the diversity of microbes. Huang et al. used the next-generation sequencing (NGS) method to prove that the ice-free area plateaus of Schirmacher Oasis contained bacteria belonging to 12 phyla and 110 genera (Huang et al., 2013), and Wang et al. reported the community structure of microorganisms sampled from different habitats (Wang et al., 2015). In summary, the incredible diversity of microorganisms in the soil of Antarctica has been reported in many studies.

Polar microorganisms are regarded as a source of cold-adapted and low-temperature enzymes and active natural products. Ray et al. isolated a strain of cold-adapted yeast, *Candida humicola*, from Schirmacher Oasis that produced high levels of protease at low temperature (Ray et al., 1992). Vazquez et al. (1995) isolated three strains of *Pseudomonas maltophilia* that displayed the highest levels of proteolytic activity at 20°C. Lario et al. (2015) analyzed a protease isolated from Antarctic algae and found that the purified protease presented optimal catalytic activity at pH 5.0 and 50°C and was stable in the presence of high concentrations of NaCl. On the other hand, an increasing number of active secondary metabolites have been found from different groups of polar microorganisms, including alkaloids, macrocyclic lipids, terpenes, peptides, quinones, polyketones and other structural types, showing antibacterial, antitumor, antiviral, immunomodulatory, antioxidant and other biological activities (Li et al., 2012; Chavez et al., 2015; Wang et al., 2016; Feng et al., 2019). These compounds with novel structures and wide activities are important lead compounds for drug research. Studies on these compounds provide a basis for the utilization of Antarctic bioresources, and the microorganisms of these studies might play important roles in future research and applications.

**FIGURE 1:**
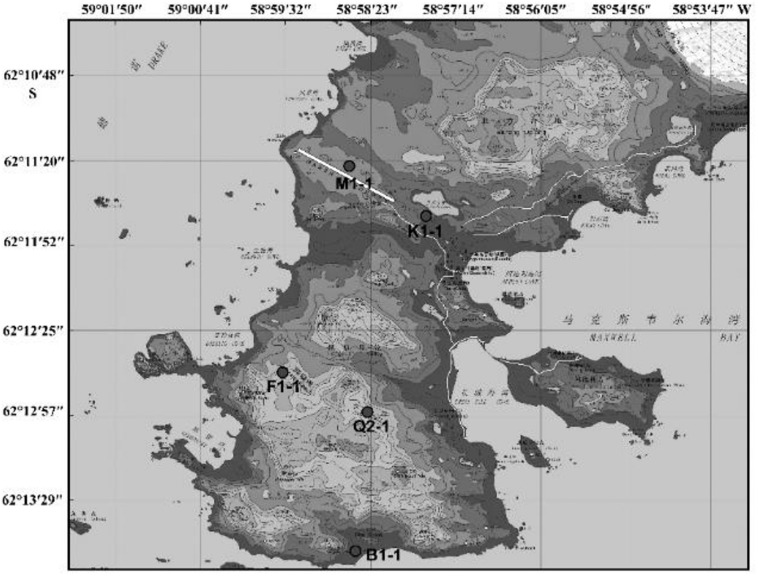
The area where sampling was performed.

Since the first Antarctic expedition in 1984, Chinese scientists have devoted themselves to the research of this continent. The biodiversity of Antarctica was one of the questions that they focused on. Using samples collected from China’s Antarctic expedition, we hoped to discover additional bacteria and fungi to increase our knowledge of the continent and identify putative enzymes and active natural products for industry and drug development. Moreover, cultivable strains can possibly be used for applications. The Fildes Peninsula is located in the southwest region of King George Island and is where the China Great Wall Station is located (Figure 1). We researched samples collected from the Fildes Peninsula, isolated and cultured microbes from soil, macroalgal rot, and sediment (Supplementary Table S1) and used 16S and ITS sequences to analyZe the evolutionary relationship of the isolated microbes. Herein, we report the isolation of 83 bacteria belonging to 20 genera and 30 fungi belonging to 7 genera from soil samples collected on the Fildes Peninsula. Furthermore, physical and chemical analyses showed that the bacteria could utilize many kinds of carbohydrates and that the fungi could produce several kinds of extracellular enzymes. At the same time, an *Aspergillus* strain named MS-19 was fermented, and four polyketones, versicone A (**1**), versicone B (**2**), 4-methyl-5,6-dihydro-2H-pyran-2-one (**3**), and (*R*)-(+)-sydowic acid (**4**), were isolated with an activity-guided approach. Among them, **1** and **2** displayed activities against *Fusarium oxysporum* and *Castanea anthracis*, especially **1**, which displayed strong activity against *Candida albicans*, with an MIC value of 3.91 μg/mL.

## Materials and Methods

### Field Site and Sampling

Samples came from the Fildes Peninsula by China’s 27th and 31st Antarctic expeditions. Soil, macroalgal rot and sediment samples were collected from Ardley Island – near the Fildes Peninsula, Antarctica. The collection location is shown in Supplementary Table S1. Sterile tools were used to collect rhizosphere soil at approximately 0–5 cm deep. Then, these soil samples were maintained at 4°C until culture and analysis. These samples were incubated at 8°C until the samples became dry. Approximately 1 g of soil was dissolved in 10 mL of sterile water and suspended. Ten-fold serial dilutions were performed, and the final concentration was 10^–5^. Then, a 100 μL suspension was spread on a plate for culture. To identify bacteria, the culture medium 2216e (peptone 5 g, yeast extraction 1 g, agar 18 g, and seawater to 1000 mL; the medium was sterilized at 121°C for 20 min, and mycostatin was added to a final concentration of 100 μg/mL) was used. For fungi, the medium PDA (potato 200 g, glucose 10 g, agar 17 g, and seawater to 1000 mL; the medium was sterilized at 121°C for 20 min, and ampicillin was added to a final concentration of 100 μg/mL) was used. The culture temperature was 12°C with incubation for 1–2 weeks. These colonies were distinguished by colony morphology and pigmentation, and distinct colonies were chosen for pure culture and preservation. Bacteria were stored at −80°C with 30% glycerin, and fungi were stored at −80°C with 20% glycerin.

### DNA Extraction, PCR Amplification and Molecular Phylogenetic Analysis

Bacteria were cultured in liquid medium for 1 week; then, the cells were collected for DNA extraction. Similarly, filtered fungi were cultured on PDA plates for 1 week. Approximately 50 mg of hyphae was ground in liquid nitrogen. DNA extraction was performed with the Genome DNA Extraction Kit (Tiangen, China). The universal primers 27F (5′-AGAGTTTGATCCTGGCTCAG-3′) and 1492R (5′- GGTTACCTTGTTACGACTT-3′) were used to amplify the bacterial 16S sequence. The reaction mixture contained 25 μL of 2 × Taq PCR MasterMix (Tiangen, China), 4 μL of both front and reverse primers, 1 μL of template DNA, and 20 μL of ddH_2_O; the total volume was 50 μL. The PCR settings were denaturation at 94°C for 5 min; 30 cycles of denaturation at 94°C for 30 s, annealing at 55°C for 30 s, and elongation at 72°C for 90 s; and a final extension at 72°C for 10 min. Agarose gel electrophoresis was employed to confirm the PCR products, and the loading amount was 5 μL. To amplify fungal DNA, the universal primers ITS1 (5′-TCCGTAGGTGAACCTGCGG-3′) and ITS4 (5′-TCCTCCGCTTATTGATATGC-3′) were used (Hughes et al., 2009). The reaction mixture contained 25 μL of 2 × Taq PCR MasterMix (Tiangen, China), 6 μL of both forward and reverse primers, 1 μL of template DNA, and 18 μL of ddH_2_O; the total volume was 50 μL. The PCR settings were denaturation at 94°C for 5 min; followed by 30 cycles of denaturation at 94°C for 30 s, annealing at 55°C for 30 s, and elongation at 72°C for 40 s; and a final extension at 72°C for 10 min. The PCR products were sent to Shanghai Sunny Biotechnology Co., Ltd. for sequencing. The 16S rDNA and ITS sequences were aligned to the GenBank database using BLAST analysis^1^. The phylogenetic tree was aligned by ClustalX, constructed by MEGA4.0 using the neighbor-joining method and visualized by the online tool iTOL^2^. Similarity comparison to type strains was completed by using the EzBioCloud Database^3^.

### Physiological-Biochemical Characteristics of Bacteria

To verify the physiology of the bacteria, the AP NE20 Kit was used according to the results of the 16S alignment. Typed strains of each genus or species were chosen for testing, and the culture temperature was set to 12°C. The protocol was provided by an API 20 NE Kit.

### Physiological-Biochemical Characteristics of Fungi

To determine these fungi abilities to produce amylase, cellulase or caseinase activities, a chosen fungus was cultured on PDA culture with 7 colonies on each plate for approximately 2 weeks at 12°C. Then, the colonies were treated using the following protocols. Amylase activity test on amylase medium: fresh iodine was added, and in the plates to stain several minutes. Then, the iodine was washed out. The colonies were checked to determine whether the surrounding area was a transparent circle. Cellulase activity test on cellulase medium: Congo red solution was added to the plate and incubated for 15 min. Then, the solution was washed out; NaCl solution was added, and the plate was incubated for 15 min. The colonies were checked to determine whether there was a transparent circle. Caseinase activity test on caseinase medium: the plate was covered with 40% trichloroacetic acid; transparency indicated a positive reaction.

### Primary Screening for Antifungal Activity

The primary screening for antifungal activity was executed using 100 × 15 mm Petri plates containing 10 mL of PDA (Hong et al., 2013). Sterile blank paper disks (0.625 cm in diameter) were placed approximately 1 cm away from a central disk of the same size. An aliquot (8 μL, 500 μg/mL) in CH_3_OH was introduced to each peripheral disk. The plates of *Fusarium oxysporum* and *Candida albicans were* incubated at 23°C for 72 h until mycelial growth from the central disk had enveloped. The peripheral disks containing the control (CH_3_OH) produced crescents indicating inhibition around the disks containing samples with antifungal activity.

### Extraction and Isolation

*Aspergillus sydowii* MS-19 was cultured on PDA plates at 20°C for 7 days. The seed medium consisting of malt extract (15 g), sea salt (10 g), and distilled water (1000 mL), pH 7.0, was inoculated with strain MS19, which was incubated at 25°C for 72 h on a rotating shaker (170 rpm). Mass scale fermentation of MS-19 was carried out using solid rice medium in 500 mL flasks (rice 80 g, sea salt 1.0 g, distilled water 80 mL) and inoculated with 10 mL of seed solution. Flasks were incubated at 20°C under a normal day night cycle. After 30 days, cultures from 20 flasks were harvested and subjected to organic extraction using ethyl acetate (EtOAc). The EtOAc extracts of solid MS19 rice medium were partitioned between petroleum ether and 90% aqueous MeOH. The resulting MeOH phase was fractionated using a silica column, Sephadex LH-20, and then semipreparative reversed-phase HPLC to obtain compounds **1**–**4** (Figure 4). The culture on solid rice medium was soaked in acetone, cut into small pieces and maintained for 1 day. The content was filtered and evaporated under vacuum using a Buchner funnel and extracted with EtOAc until exhaustion; this process was repeated three times. The organic phase was collected and evaporated, and a dark brown oil crude extract (13.5 g) was obtained. The crude EtOAc extract was subjected to silica gel column chromatography (CC) eluted with petroleum ether/EtOAc in a gradient (v/v, 50:1, 30:1, 20:1, 10:1, 5:1, 1:1, 0:1), and 8 fractions (fractions 1–8) were obtained on the basis of TLC. Fr. 3 was purified by CC (petroleum ether/EtOAc, 5:2) to give 6 subfractions (fr. 3.1–3.6). Fr. 3.3 was further purified by (SP-RP) HPLC eluting with CH_3_OH-H_2_O (80:20) to afford compound **2** (4.5 mg). Fr. 4 was further purified by Sephadex LH-20 (petroleum ether/CHCl_3_/MeOH, 5:2:1) to give 6 subfractions (fr. 4.1–4.6). Fr. 4.3 was further purified by (SP-RP) HPLC eluting with CH_3_CN-H_2_O (60:40, 1‰ TFA) to afford compounds **3** (4.0 mg) and **4** (38.3 mg). Compound **1** (3.7 mg) was isolated from fr. 4.2 by (SP-RP) HPLC using 80% MeCN.

### Antifungal Activity Assay

The antifungal activity assay was performed using the broth microdilution method (Wang et al., 2016). Arrayed stock solutions of the tested compounds dissolved in DMSO were diluted 100-fold with the proper culture medium for each pathogenic fungus, and preliminary screening was carried out under sterile conditions with 500 μg/mL (the highest concentration). Under a sterile environment, fungal suspensions (50 μL) of each pathogenic fungus were poured into wells containing 50 μL of 2-fold serially diluted single compounds in the corresponding culture medium for a final volume of 100 μL. The negative controls were treated with 1% DMSO. At the same concentrations, blank wells were prepared with the corresponding culture medium containing the tested compounds. The inoculated plates were incubated at 28°C. After incubation for 48 h, the optical density (OD) of each well was measured using a microplate reader at 600 nm. The minimum inhibitory concentration (MIC) values were derived from Probit analysis of the concentration, response data, with serially diluted concentrations of the tested compounds. The dilutions of the tested compounds were performed three times. Amphotericin B was used as a positive control against two fungi (*Fusarium oxysporum* and *Candida albicans*) with MIC values of 1.25 and 0.625 μg/mL, respectively.

## Results and Discussion

### Diversity and Phylogeny of Soil Microorganisms

According to the cultivable results, we identified 83 strains of bacteria and 30 strains of fungi from soil, macroalgal rot and sediment samples collected on the Fildes Peninsula, Antarctica. By using 16S and ITS sequence similarity alignments, we identified 33 apparently different species of bacteria belonging to 20 genera and 4 phyla and 8 species of fungi belonging to 6 genera and 4 classes. The 33 species of bacteria belonged to *Proteobacteria* (23), *Firmicutes* (1), *Actinobacteria* (5), and *Bacteroidetes* (4). The dominant bacterial genus was *Pseudomonas* (7). Distinct differences were observed among the 5 samples. The widespread genera *Pseudomonas* and *Massilia* belonged to *Proteobacteria*, while *Arthrobacter* belonged to *Actinobacteria*. *Pseudomonas* and *Massilia* were detected in samples F1-1, K1-1, M1-1, and Q2-1, while *Arthrobacte*r was detected in B1-1, F1-1, K1-1 and M1-1. Usually, we regarded a subject as a potential novel species if the 16S sequence similarity was < 97% (McCaig et al., 1999). From the 33 strains, we suggested that 1 may be novel species because of their low similarity, which is shown in Table 3. All the sequences we obtained have been submitted to GenBank, and their accession numbers are shown in Tables 1, 2.

**TABLE 1:** Identification of culturable bacteria isolated from the soil of the Fildes Peninsula.

Genus	Stain no.	Most similar strain (ID)	Similarity	GenBank accession no.
*Pseudomonas*	1PF3	*Pseudomonas arsenicoxydans* VC-1(FN645213)	99.85%	KT991031
	1PQ2-6	*Pseudomonas migulae* CIP 105470(AF074383)	99.70%	KT991032
	1K1lan	*Pseudomonas migulae* CIP 105470(AF074383)	99.71%	KT991033
	1Q1lan-6	*Pseudomonas migulae* CIP 105470(AF074383)	99.71%	KT991034
	1EK3	*Pseudomonas migulae* CIP 105470(AF074383)	99.63%	KT991035
	1EQ1	*Pseudomonas migulae* CIP 105470(AF074383)	99.71%	KT991036
	1PK1	*Pseudomonas migulae* CIP 105470(AF074383)	99.71%	KT991037
	W3-1-3	*Pseudomonas migulae* CIP 105470(AF074383)	99.63%	KT991038
	2EK3	*Pseudomonas migulae* CIP 105470(AF074383)	99.63%	KT991039
	2PK7	*Pseudomonas graminis* DSM 11363(Y11150)	99.73%	KT991040
	2EK4	*Pseudomonas graminis* DSM 11363(Y11150)	99.78%	KT991041
	1F1lan	*Pseudomonas mandelii* CIP 105273(AF058286)	99.41%	KT991042
	1PM2	*Pseudomonas mandelii* CIP 105273(AF058286)	99.41%	KT991043
	1PF2-6	*Pseudomonas mandelii* CIP 105273(AF058286)	99.11%	KT991044
	1PK2	*Pseudomonas mandelii* CIP 105273(AF058286)	99.78%	KT991045
	2PM9	*Pseudomonas mandelii* CIP 105273(AF058286)	99.64%	KT991046
	1PM1	*Pseudomonas avellanae* BPIC631(AKBS01001374)	99.11%	KT991047
	1EK4-6	*Pseudomonas avellanae* BPIC631(AKBS01001374)	99.05%	KT991048
	1PM3	*Pseudomonas avellanae* BPIC631(AKBS01001374)	99.13%	KT991049
	2M2lan	*Pseudomonas avellanae* BPIC631(AKBS01001374)	99.12%	KT991050
	2PF3	*Pseudomonas avellanae* BPIC631(AKBS01001374)	98.46%	KT991051
	2PF2lan	*Pseudomonas avellanae* BPIC631(AKBS01001374)	99.13%	KT991052
	2PM4	*Pseudomonas avellanae* BPIC631(AKBS01001374)	99.05%	KT991053
	2PQ2	*Pseudomonas frederiksbergensis* JAJ28(AJ249382)	99.78%	KT991054
	2EK2	*Pseudomonas frederiksbergensis* JAJ28(AJ249382)	98.77%	KT991055
	Q1-3-2	*Pseudomonas frederiksbergensis* JAJ28(AJ249382)	99.78%	KT991056
	Q1-3-1	*Pseudomonas frederiksbergensis* JAJ28(AJ249382)	99.78%	KT991057
	1EQ2	*Pseudomonas meridiana* CMS 38(AJ537602)	99.41%	KT991058
	Q2-1-2	*Pseudomonas meridiana* CMS 38(AJ537602)	99.41%	KT991059
	2EM1	*Pseudomonas meridiana* CMS 38(AJ537602)	99.56%	KT991060
	2PQ3	*Pseudomonas meridiana* CMS 38(AJ537602)	99.49%	KT991061
	2PQ41	*Pseudomonas meridiana* CMS 38(AJ537602)	99.49%	KT991062
	2EQ1	*Pseudomonas meridiana* CMS 38(AJ537602)	99.56%	KT991063
	2EM5	*Pseudomonas meridiana* CMS 38(AJ537602)	99.56%	KT991064
*Neptunomonas*	1EF2	*Naphthovorans* NAG-2N-126(AF053734)	97.81%	KT991065
*Psychrobacter*	1EB1	*Psychrobacter glacincola* DSM 12194(AJ312213)	99.19%	KT991066
	1EB2	*Psychrobacter glacincola* DSM 12194(AJ312213)	99.49%	KT991067
	2EB3	*Psychrobacter glacincola* DSM 12194(AJ312213)	99.42%	KT991068
	2EB4	*Psychrobacter glacincola* DSM 12194(AJ312213)	99.49%	KT991069
	2EB5	*Psychrobacter glacincola* DSM 12194(AJ312213)	99.20%	KT991070
	2EB11	*Psychrobacter glacincola* DSM 12194(AJ312213)	99.20%	KT991071
	2EB12	*Psychrobacter glacincola* DSM 12194(AJ312213)	99.20%	KT991072
	2EB21	*Psychrobacter glacincola* DSM 12194(AJ312213)	99.20%	KT991073
	2PB1lan	*Psychrobacter urativorans* DSM 14009(AJ609555)	99.93%	KT991074
*Luteibacter*	2PK1	*Luteibacter rhizovicinus* LJ96(AJ580498)	97.77%	KT991075
	2PK6	*Luteibacter rhizovicinus* LJ96(AJ580498)	97.67%	KT991076
	2PK8	*Luteibacter rhizovicinus* LJ96(AJ580498)	97.75%	KT991077
*Burkholderia*	2PQ1	*Burkholderia udeis* LMG 27134(AY154367)	98.81%	KT991078
*Massilia*	2PK9	*Massilia aurea* AP13(AM231588)	98.01%	KT991079
	2PF3lan	*Massilia plicata* 76(AY966000)	98.17%	KT991080
	2PM4lan	*Massilia eurypsychrophila* B528-3(KJ361504)	99.33%	KT991081
	2PQ5	*Massilia eurypsychrophila* B528-3(KJ361504)	99.33%	KT991082
	2PM3lan	*Massilia eurypsychrophila* B528-3(KJ361504)	99.33%	KT991083
*Rugamonas*	1K2lan	*R*μ*gamonas rubra* MOM 28/2/79(HM038005)	98.57%	KT991084
*Janthinobacterium*	K1-1	*Janthinobacterium svalbardensis* JA-1(DQ355146)	99.85%	KT991085
*Duganella*	2M1lan	*Duganella phyllosphaerae* T54(FR852575)	97.13%	KT991086
*Planomicrobium*	0.1a	*Planomicrobium okeanokoites* IFO 12536(D55729)	99.83%	KT991087
	1a	*Planomicrobium okeanokoites* IFO 12536(D55729)	99.83%	KT991088
*Rhizobium*	2PK3	*Rhizobium tubonense* CCBAU 85046(EU256434)	99. 08%	KT991089
	2PK7lan	*Rhizobium tubonense* CCBAU 85046(EU256434)	99. 80%	KT991090
*Brevundimonas*	N1-1-1	*Brevundimonas bullata* IAM 13153(D12785)	100%	KT991091
*Sphingomonas*	1PM7	*Sphingomonas aerolata* NW12(AJ429240)	99.32%	KT991092
	2PF4	*Sphingomonas glacialis* C16y(GQ253122)	99.77%	KT991093
	2PM11	*Sphingomonas glacialis* C16y(GQ253122)	99.85%	KT991094
*Sulfitobacter*	1EK2	*Sulfitobacter pontiacus* DSM 10014(Y13155)	99.77%	KT991095
*Streptomyces*	2PM10	*Streptomyces avidinii* NBRC 13429(AB184395)	99.77%	KT991096
*Microterricola*	1EM1	*Microterricola viridarii* KV-677(AB282862)	99.33%	KT991097
	2EM3	*Microterricola viridarii* KV-677(AB282862)	99.33%	KT991098
*Arthrobacter*	1EB3	*Arthrobacter antarcticus* SPC26(AM931709)	99.11%	KT991099
	1EF3-6	*Arthrobacter ginsengisoli’* DCY81(KF212463)	99.41%	KT991100
	D1-1-1	*Arthrobacter ginsengisoli’* DCY81(KF212463	99.48%	KT991101
	F1-1-1	*Arthrobacter ginsengisoli’* DCY81(KF212463)	99.33%	KT991102
	2PF1lan	*Arthrobacter ginsengisoli’* DCY81(KF212463)	99.63%	KT991103
	2EF1	*Arthrobacter ginsengisoli’* DCY81(KF212463)	99.48%	KT991104
	2EK1	*Arthrobacter ginsengisoli’* DCY81(KF212463)	99.41%	KT991105
	2EM2	*Arthrobacter ginsengisoli’* DCY81(KF212463)	99.48%	KT991106
	1M2lan	*Arthrobacter psychrochitiniphilus* GP3(AJ810896)	98.94%	KT991107
	1EM2	*Arthrobacter psychrochitiniphilus* GP3(AJ810896)	98.96%	KT991108
	M1-1-2	*Arthrobacter psychrochitiniphilus* GP3(AJ810896)	98.96%	KT991109
*Gillisia*	2EM4	*Gillisia hiemivivida* IC154(AY694006)	99.33%	KT991110
*Pedobacter*	2PM12	*Pedobacter panaciterrae* Gsoil 042(AB245368)	97.72%	KT991111
*Mucilaginibacter*	2PM7	*Mucilaginibacter dorajii* DR-f4(GU139697)	96.52%	KT991112
	2PK12	*Mucilaginibacter soli* R9-65(JF701183)	97.64%	KT991113

**TABLE 2:** Identification of culturable fungi isolated from the soil of the Fildes Peninsula.

Genus	Strain no.	Most similar strain (ID)	Similarity	GenBank accession no.
*Geomyces*	1PM6	*Geomyces pannorum* UFMGCB6110(KC485453)	100%	KT991114
	1EK1	*Geomyces pannorum* UFMGCB6110(KC485453)	98.10%	KT991115
	2PB5	*Geomyces pannorum* UFMGCB6110(KC485453)	100%	KT991116
	2PM1	*Geomyces pannorum* UFMGCB6110(KC485453)	100%	KT991117
	1PF2-8	*Geomyces pannorum* UFMGCB6049(KC485437)	100%	KT991118
	1PQ2-8	*Geomyces pannorum* UFMGCB6049(KC485437)	99.06%	KT991119
	1Q1lan-8	*Geomyces pannorum* UFMGCB6049(KC485437)	99.53%	KT991120
	1PQ1	*Geomyces pannorum* UFMGCB6049(KC485437)	100%	KT991121
	1EF3-8	*Geomyces pannorum* UFMGCB6049(KC485437)	100%	KT991122
	1PB1	*Geomyces pannorum* UFMGCB6049(KC485437)	100%	KT991123
	1	*Geomyces pannorum* UFMGCB6049(KC485437)	100%	KT991124
	4	*Geomyces pannorum* UFMGCB6049(KC485437)	100%	KT991125
	6	*Geomyces pannorum* UFMGCB6049(KC485437)	100%	KT991126
	2PB3	*Geomyces pannorum* UFMGCB6049(KC485437)	100%	KT991127
	2PB4	*Geomyces pannorum* UFMGCB6049(KC485437)	100%	KT991128
	2PB1	*Geomyces pannorum* UFMGCB6049(KC485437)	100%	KT991129
	2PB2	*Geomyces pannorum* UFMGCB6049(KC485437)	100%	KT991130
	2PQ6	*Geomyces pannorum* UFMGCB6049(KC485437)	99.76%	KT991131
	2PF1	*Geomyces pannorum* Geo-6(JF320819)	100%	KT991132
*Pseudeurotium*	W2-1	*Pseudeurotium desertorum* WL07-3(JX077048)	96.46%	KT991133
	2PM2	*Pseudeurotium desertorum* WL07-3(JX077048)	96.46%	KT991134
*Rhizoscyphus*	2PK2	*Rhizoscyphus monotropae* ATCC52305(AF169309)	91.82%	KT991135
*Lecythophora*	2PQ42	*Lecythophora fasciculata* IFM 50359(KT991136)	96.58%	KT991136
*Rhodotorula*	2PK13	*Rhodotorula rosulata* CBS10977(EU872492)	89.59%	KT991137
*Mortierella*	1PM5	*Mortierella elongatula* CBS 488.70(HQ630349)	95.91%	KT991138
	1EK4-8	*Mortierella elongatula* CBS 488.70(HQ630349)	95.12%	KT991139
	1PM4	*Mortierella elongatula* CBS 488.70(HQ630349)	95.91%	KT991140
	2EB6	*Mortierella elongatula* CBS 488.70(HQ630349)	95.91%	KT991141
	2EK5	*Mortierella elongatula* CBS 488.70(HQ630349)	95.91%	KT991142
*Aspergillus*	MS-19	*Aspergillus sydowii DBOF 102* (JQ724463)	99.13%	JX675047

Fungi identification revealed 6 genera belonging to 4 classes: *Mortierella (Mortierellomycotina), Geomyces, Pseudeurotium, Hymenoscyphus (Leotiomycetes)*, Lecythophora (*Sordariomycetes*), *Rhodotorula* (*Microbotryomycetes*), and *Aspergillus*. The most abundant fungal genus was *Geomyces*. Several fungal strains with significant differences compared to typical strains were found. Usually, the subject was regarded as a potential novel species if the ITS sequence similarity was < 97% (Hughes et al., 2009). It was suggested that these 6 strains include 3 potential novel species might belong to 2 genera. From the results, we found that the microbes from the Fildes Peninsula, Antarctica, were plentiful although some of them never having been reported previously and should be investigated further (Table 3).

**TABLE 3:** Similarity comparison of all the sequences of potential novel species.

Microbial type	Strain no.	Most similar strain (ID)	Similarity
Bacteria	2PM7	*Mucilaginibacter dorajii* DR-f4(GU139697)	96.52%
Fungi	W2-1, 2PM2	*Pseudeurotium desertorum* WL07-3(JX077048)	96.46%
	1PM5, 1PM4, 2EB6, 2EK5	*Mortierella elongatula* CBS 488.70(HQ630349)	95.91%
	1EK4-8	*Mortierella elongatula* CBS 488.70(HQ630349)	95. 12%
	2PQ42	*Lecythophora fasciculata* IFM 50359 (KT991136)	96.58%
	2PK2	*Rhizoscyphus monotropae* ATCC52305(AF169309)	91.82%
	2PK13	*Rhodotorula rosulata* CBS10977(EU872492)	89.59%

**FIGURE 2:**
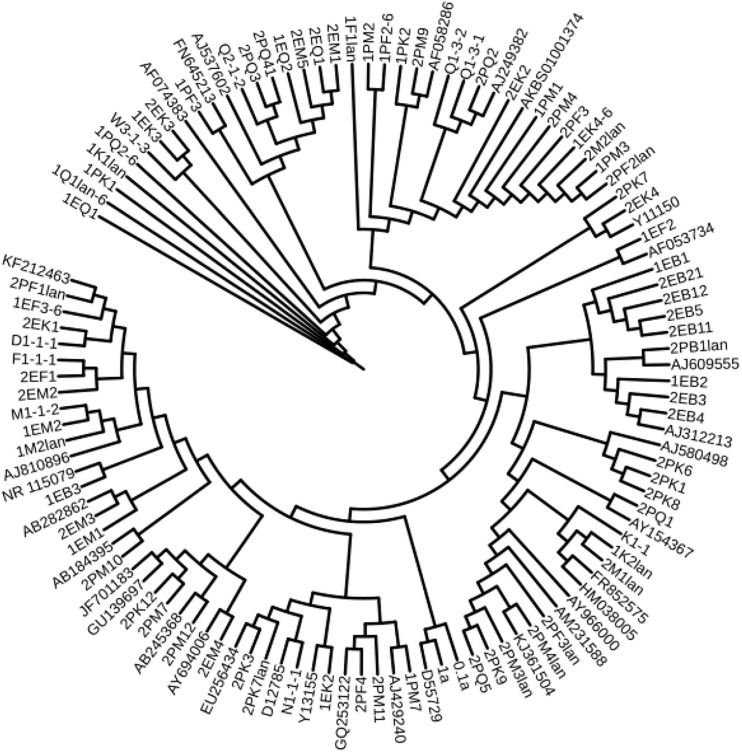
Antarctica bacteria phylogenetic trees based on 16S rDNA sequences.

**FIGURE 3:**
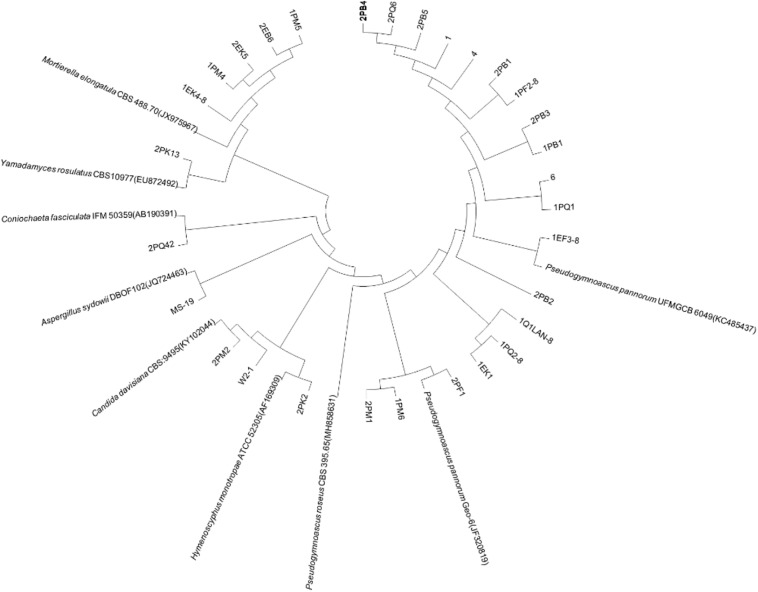
Antarctica fungus phylogenetic trees based on ITS sequences.

We used the neighbor-joining method to construct a phylogenetic tree according to the similarity of 16S and ITS sequences. The bootstrap value of each branch is the result of 1000 replications. The trees are shown in Figures 2, 3.

### Physiological-Biochemical Characteristics of Bacteria

Typical and putative novel strains were chosen from the cultured bacteria, and physical and chemical analyses were performed using the API NE20 Kit (bioMérieux). As shown in Table 4, 30, 23, 24, and 8 strains of them could produce α-glucosidase, protease, urease and β-galactosidase, respectively. The tested bacteria displayed the ability to utilize a broad spectrum of organics. Strain 2EK2 could reduce nitrate to nitrite and nitrogen, produce arginine dihydrolase, urease, α-glucosidase and protease, and assimilate glucose, mannitol, potassium gluconate, capric acid, adipic, acid, malate and sodium citrate. 2PK7 could reduce nitrate to nitrite and nitrogen, produce arginine dihydrolase, urease, α-glucosidase, and β-galactosidase, and assimilate glucose, arabinose, mannose, mannitol, acetylglucosamine, potassium gluconate, capric acid, malate, and sodium citrate; 2PM3lan could reduce nitrate to nitrogen, produce α-glucosidase and protease and assimilate all 12 kinds of substrates tested. 2PF3lan could produce arginine dihydrolase, urease, α-glucosidase, and β-galactosidase and assimilate glucose, mannose, acetylglucosamine, maltose, potassium gluconate, malate, sodium citrate, and phenylacetic acid (Table 4).

**TABLE 4:** API physiological and biochemical characterization of culturable bacteria.

Indicators	NO_3_	TRP	GLU	ADH	URE	ESC	GEL	PNP	GLU	ARA	MNE	MAN	NAG	MAL	GNT	CAP	ADI	MLT	CIT	PAC
1PF3	−	−	±	+	+	+	+	−	+	+	+	+	+	−	+	+	−	+	+	−
1PQ2	−	−	−	+	+	+	+	−	+	+	+	+	+	±	+	+	±	+	+	±
2PK7	−	−	−	±	+	+	−	+	+	±	+	+	+	−	+	+	−	+	+	−
1PM2	+	−	−	−	+	+	+	−	−	−	−	−	−	−	−	−	−	−	+	−
1PM3	+	−	−	−	+	+	+	−	−	−	−	−	−	−	−	−	−	−	±	−
2EK2	+	−	−	±	±	+	+	−	+	−	−	+	−	−	+	+	±	+	+	−
2EM1	−	−	−	−	−	−	+	−	+	+	+	+	+	−	+	+	−	+	+	−
1EF2	+	−	−	−	+	+	+	−	−	−	−	−	−	−	−	−	±	−	+	−
2EB3	−	−	−	−	−	−	±	−	−	−	−	−	−	−	−	−	−	−	−	−
2PB1lan	+	−	−	−	+	+	+	−	−	−	−	−	−	−	−	−	−	−	−	−
2PK8	+	−	−	−	+	−	−	+	−	−	−	−	−	−	−	−	−	±	±	−
2PQ1	−	−	−	+	+	+	−	−	+	−	−	−	−	−	−	−	+	−	−	−
2PK9	−	−	−	−	+	+	−	−	−	−	−	−	−	−	−	−	−	−	+	−
2PF3lan	−	−	−	±	+	+	+	+	+	−	+	+	+	+	+	−	−	+	+	+
2PM3lan	+	−	−	−	−	+	+	−	+	+	+	+	+	+	+	+	+	+	+	±
1K2lan	+	−	−	+	+	+	−	−	−	−	−	−	−	−	−	−	±	±	−	−
K1-1	−	−	−	−	−	+	−	−	−	−	−	−	−	−	−	−	−	+	−	−
2M1lan	−	−	−	+	+	+	−	+	−	−	−	−	−	−	−	−	−	±	−	−
0.1a	+	−	−	−	−	+	+	−	+	−	−	−	−	−	−	−	−	−	−	±
2PK3	−	−	−	−	−	+	−	+	+	−	−	−	+	+	−	−	−	−	−	−
N1-1−1	+	−	+	+	+	+	−	+	+	−	+	+	+	+	+	+	−	+	+	+
1PM7	+	−	+	+	+	+	+	−	+	+	+	+	+	−	+	+	−	+	+	−
2PM11	−	−	−	−	+	+	+	−	−	−	−	+	+	−	+	−	−	+	+	−
1EK2	−	−	−	−	−	+	+	−	−	−	−	−	−	−	−	−	−	−	−	−
2PM10	+	−	−	−	+	+	+	−	−	−	−	−	−	−	−	−	−	−	+	−
1EM1	+	−	−	−	+	+	+	−	−	−	−	−	−	−	−	−	−	−	+	−
1EB3	+	−	−	−	+	+	+	−	−	−	−	+	−	−	−	+	−	+	+	−
2EF1	+	−	−	−	−	+	+	−	−	−	±	±	−	−	−	−	+	+	±	−
1EM2	+	−	−	−	+	+	+	−	−	−	−	−	−	−	−	−	±	±	−	−
2EM4	−	−	−	−	−	+	+	−	−	−	−	−	−	−	−	−	−	−	−	−
2PM12	+	−	−	−	+	+	±	−	±	±	−	−	−	−	−	−	−	−	+	−
2PM7	−	−	−	−	+	+	−	+	+	−	+	+	+	+	+	−	±	+	−	±
2PK12	−	−	+	+	+	+	+	+	+	+	+	+	+	±	+	+	±	+	+	+

*(***1****) N03, TRP, GLU, ADH, URE, ESC, GEL, and PNG represent nitrate reduction, indole synthesis, glucose fermentation, arginine dihydrolysis, urease, hydrolysis by α*−*glucosidase, protease and β*−*galactosidase, respectively.* (***2***) *GLU, ARA, MNE, MAN, NAG, MAL, GNT, CAP, ADI, MLT, CIT and PAC represent glucose, arabinose, mannose, mannitol, acetylglucosamine, maltose, potassium gluconate, capric acid, adipic acid, malate, sodium citrate and phenylacetic acid, respectively. (****3****) “+” represents positive reaction, “*−*” represents negative reaction, “±” represents weakly positive reaction.**

### Detection of Ectoenzyme Activities in Fungi

Extracellular enzymes were detected from typical and putative novel strains chosen from the culturable fungi. Results revealed that 7 strains were positive for amylase activity, 6 strains were positive for cellulase activity, and 10 strains were positive for caseinase activity. Six strains showed the abilities to produce all three enzymes (Table 5). The proportions of the strains encoding amylase, cellulase and caseinase were 53.85, 46.15, and 76.92%, respectively.

**TABLE 5:** Extracellular enzyme activity of culturable fungi.

Stain no.	Amylase	Cellulase	Caseinase
2PM1	−	−	−
1PQ1	+	+	+
1PQ2	+	+	+
2PF1	+	+	+
W2-1	−	−	±
2PK2	−	−	+
2PQ42	−	−	±
2PK13	+	−	+
1PM5	+	+	+
1EK4-8	−	−	−
1PM4	+	+	+
2EB6	+	±	+
2EK5	−	−	−

**“+” represents positive, “*− *” represents negative, “±” represents weakly positive.**

### Primary Screening for Antifungal Activity

The EtOAc extract of rice fermentation of 30 fungi was prepared, and the antifungal activities of the 30 strains were screened by the filter paper method (Hong et al., 2013). A preliminary screening revealed that only the fungus MS-19, identified as *Aspergillus sydowii*, was able to inhibit the growth of 2 pathogenic fungi (*F.oxysporum* and *C. albicans*). The neighbor-joining tree and morphological and microscopic characteristics of MS-19 are shown in Supplementary Figure S1 and in previous literature (Cong et al., 2017).

### Compound Identification

**FIGURE 4:**
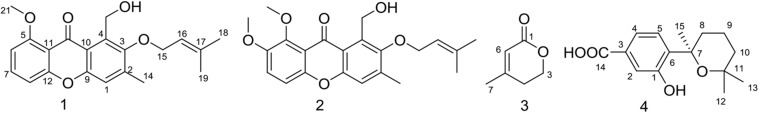
Structure of compounds **1–4.**

The EtOAc extract of rice fermentation of *Aspergillus sydowii* MS-19 was subjected to silica gel column chromatography and further purified by HPLC to obtain four known compounds (**1–4**) (Figure 4). The spectroscopic data of the identified compounds were compared with those reported in the literature, and versicone A (**1**), versicone B (**2**), 4-methyl-5,6-dihydro-2H-pyran-2-one (**3**), and (*R*)-(+)-sydowic acid (**4**) were identified. Compounds **1–3** were isolated from *Aspergillus sydowii* for the first time.

Compound **1** was obtained as a yellow amorphous solid. The ^1^H NMR spectrum showed the presence of 3 methyl groups [δ_H_ 1.78 (s, H_3_-18), δ_H_ 1.70 (s, H_3_-19), δ_H_ 2.41 (s, H_3_-14),], one methoxy group [δH 4.02 (s, H_3_-21)], and 4 olefinic protons [δ_H_ 7.23 (s, H-1), δ_H_ 6.79, (d, *J* = 8.5 Hz, H-6), δ_H_ 7.58 (t, *J* = 8.5 Hz, H-7), δ_H_ 7.01 (dd, *J* = 8.5, 0.5 Hz, H-8)]. Analysis of the ^13^C NMR data of **1** revealed 21 carbon signals, involving 3 methyl groups (δ_C_ 17.7, CH_3_-14; δ_C_ 18.3, CH_3_-19_;_ δ_C_ 26.0, CH_3_-18), one methoxy group (δ_C_ 56.8, CH_3_-21), 2 oxygenated methylenes (δ_C_ 57.3, CH_2_-20; δ_C_ 72.3, CH_2_-15), 5 olefinic methines (δ_C_ 109.9, CH-8; δ_C_ 105.6, CH-6; δ_C_ 119.1, CH-1; δ_C_ 120.1, CH-16; δ_C_ 134.7, CH-7), 9 quaternary carbons including 4 oxygenated carbons (δ_C_ 152.9, C-3; δ_C_ 160.8, CH-5; δ_C_ 152.8, C-9; δ_C_ 157.6, C-12), and one carboxyl carbon (δ_C_ 179.9, C-13) (Supplementary Table S2). These signals were exactly the same as those of versicone A (Wang et al., 2016).

The resemblance of the ^1^H and ^13^C NMR data (Supplementary Table S2) of **2** and **1** indicated that they had the same skeleton. The main difference in the ^1^H NMR spectra was the presence of a methoxy group at δ_H_ 3.89 in **2**, and in the ^13^C NMR spectrum, a methoxy group at δ_C_ 57.4 in **2**. Therefore, **2** was presumed to be a product of the methylation of 1 at C-6 (δ_C_ 105.6 in **1**, δ_C_ 149.1 in **2**). This conclusion was supported because the NMR data of **2** was identical to that of versicone B. Versicones A-B were first discovered from the culture medium of *Aspergillus versicolour* SCSIO 05879 (Wang et al., 2016).

Compound **3** was isolated as a colorless oil. The ^1^H NMR and ^13^C NMR spectra showed the presence of one methyl group (δ_H_ 2.01, δ_C_ 22.6, CH_3_-7), one methylene group [δ_H_ 2.38 (t, *J* = 6.0 Hz), δ_C_ 29.2, CH_2_-4], one oxygenated methylene [δ_H_ 4.38 (t, *J* = 6.0 Hz), δ_C_ 65.9, CH_2_-3], one methine [δ_H_ 5.83 (q = 1.5 Hz), δ_C_ 116.8, CH-6], and two quaternary carbons (δ_C_ 157.8, C-5; δ_C_ 164.8, C-1) (Supplementary Table S3). Through literature comparison, **3** was identified as 4-methyl-5,6-dihydro-2H-pyran-2-one (Hamasaki et al., 1975).

Compound **4** forms colorless needles, [α]25 D + 13.6 (*c* 0.20, CHCl_3_). The NMR spectra indicated the presence of 20 protons and 15 carbons, including 3 methyls, 3 methylenes, 3 methine, and 6 quaternary carbons (Supplementary Table S4). Based on these data and the literature, **4** could easily be identified as sydowic acid (Ying et al., 2014).

### Antifungal Activities

The antifungal activities of compounds **1–4** against two pathogenic fungi (*Fusarium oxysporum*, *Candida albicans*) were preliminarily investigated. Amphotericin B was used as the corresponding positive control. Among them, **1** showed strong antifungal activity (MIC of 3.91 μg/mL) against *Candida albicans* compared with amphotericin B (MIC of 0.625 μg/mL). In addition, the antifungal activities of other compounds were not ideal (Table 6).

**TABLE 6:** Inhibitory effects of compounds **1–4** on pathogenic fungi.

Compound	Pathogenic fungi (MIC, μg/mL)	
	*Fusarium oxysporum*	*Candida albicans*
1	62.5	3.91
2	>500	125
3	>500	>500
4	>500	>500
Amphotericin B	1.25	0.625

## Conclusion

To better understand the biodiversity and the potential application of microbes living in Antarctica, we collected, isolated, cultured and analyzed the composition and function of microorganisms located on the Fildes Peninsula, Antarctica. Different kinds of culture media satisfy different microbial preferences, so we cultured the microbes in several media to obtain more species.

The bacteria we isolated from the soil collected on the Fildes Peninsula, Antarctica, included 33 species belonging to 4 phyla, *Proteobacteria* (23), *Firmicutes* (1), *Actinobacteria* (5), and *Bacteroidetes* (4), in 20 genera. A total of 7 species belonging to *Pseudomonas*, the dominant genus, were isolated, and 5 species belonging to *Arthrobacter* were obtained. This result was in accordance with Ding et al., who reported that the dominant genus belonged to *Gammaproteobacteria* (Ding et al., 2014). On the other hand, Dong et al. reported that the dominant genus belonged to the phylum Firmicutes (Dong et al., 2013). Compared with the *pyrosequencing* results from previous studies (Wang et al., 2015), many phyla were not detected, including *Acidobacteria* and *Verrucomicrobi*a. This might be because it is difficult to culture cells of these phyla (Hedlund, 2010; Thrash and Coates, 2010).

We isolated a fewer number of fungi than bacteria. Only 7 genera were recognized, *Mortierella*, *Geomyces*, *Hymenoscyphus*, *Pseudeurotium*, *Lecythophora Rhodotorula*, and *Aspergillus*, and the most common genus was *Geomyces*. Factors such as the variance of sampling places, sampling seasons, culture media, and methods might contribute to these differences. All 6 fungal genera we identified belonged to *Ascomycota* and *Basidiomycota*, and the results were consistent with those of Dong et al. (2015). In conclusion, we suggested that the microbes of the Fildes Peninsula are diverse; these results exceeded our estimation and provided a source for researching metabolism and biodegradation.

Among the isolated bacteria and fungi, 3 bacteria and five fungi had less similarity to references than others, suggesting that the 11 strains may be novel species. Usually, strains with 16S rDNA and ITS sequence similarity less than 97% could be regarded as novel species (McCaig et al., 1999; Hughes et al., 2009). Dong et al. reported several potential novel species (Dong et al., 2013), and one of them was verified as *Deinococcus antarcticus* sp. nov. (Dong et al., 2015). Although these putative novel species need to be further identified, this information could enrich our knowledge of Antarctica. To date, scientists have proven that many psychotropic and cold-resistant bacteria isolated from polar regions can synthesize enzymes that function at low temperatures. β-Galactosidase plays an important role in the degradation of cellulose (Pardee et al., 1959), and the strains detected in our study, including 2PK7, 2PK8, 2PF3lan, 2M1lan, 2PK3, N1-1-1, 2PM7, and 2PK12, could produce β-galactosidase. Previous reports have also shown that other fungal genera can synthesize β-galactosidase. Ding et al. obtained 2 strains of bacteria from Prydz Bay, Antarctica, that belonged to Microbacterium and Salegentibacter and could synthesize β-galactosidase (Ding et al., 2014). Turkiewicz et al. reported a novel species, *Thysanoessa macrura*, from the alimentary tract of Antarctic krill; this species could synthesize an intracellular cold-adapted β-galactosidase (Turkiewicz et al., 2003). All these strains that could synthesize β-galactosidase may provide candidates for industrial applications.

Macromolecules were utilized by microbes after hydrolysis (Thomas, 1980; Confer and Logan, 1997). By hydrolysing α-1,4-glycosidic bonds, α-glucosidase can produce glucose from polyoses such as amylose (der Maarel et al., 2002). Therefore, α-glucosidase plays an important role in the utilization of carbohydrates (Elbein, 1991). The strains 1PF3, 1PQ2, 2PK7, 1PM2, 2PB1lan, 2PF3lan, 2PM3lan, 2PM11, and EB3 could produce α-glucosidase, indicating that these strains were able to degrade polyose.

The tested strains showed broad-spectrum utilization of multiple kinds of carbohydrates. For example, 2EK2 could assimilate glucose, mannitol, potassium gluconate, capric acid, adipic acid, malate and sodium citrate; 1PF3, 2PK7, 2EM1, and 1PM7 could assimilate glucose, arabinose, mannose, mannitol, acetylglucosamine, potassium gluconate, capric acid, malate, and sodium citrate. N1-1-1 could assimilate glucose, mannose, mannitol, acetylglucosamine, maltose, potassium gluconate, capric acid, malate, sodium citrate and phenylacetic acid; 1PQ2, 2PM3lan, and 2PK12 could utilize all 12 kinds of carbohydrates tested. Therefore, these bacteria are substantial candidates for low-temperature applications.

The isolated fungi were tested to determine whether they could produce extracellular enzymes. The results showed that 7 of 13 had amylase activity, 6 of 13 had cellulase activity, and 10 of 13 had caseinase activity. Six strains displayed all three enzyme functions. Cellulase, amylase, and caseinase have important applications in industry (Souza, 2010; Kuhad et al., 2011; Shrestha et al., 2015). Because the testing temperature was set to 12°C, the testing results suggested that these Antarctic fungi had adapted to the extreme environment and may be a repository of low-temperature working enzymes. Former studies have provided suggestions for the utilization of biocatalysts (Robl et al., 2013).

In this paper, the antifungal activities of 30 fungi were also evaluated. As a result, the fungus MS-19, identified as *Aspergillus sydowii*, was able to inhibit the growth of *Castanea anthracis* and *Fusarium oxysporum*. Further isolation yielded four polyketones: versicone A (**1**), versicone B (**2**), 4-methyl-5,6-dihydro-2H-pyran-2-one (**3**), and (*R*)-(+)-Sydowic Acid (**4**). Among them, versicone A displayed strong activity against *Castanea anthracis* with an MIC value of 3.91 μg/mL. The results indicated that versicone A could be regarded as a lead compound against *Candida albicans*.

## Data Availability Statement

The datasets presented in this study can be found in online repositories. The names of the repository/repositories and accession number(s) can be found in the article/ Supplementary Material.

## Author Contributions

BC, XY, and AD contributed to isolation and identification of microbes of Antarctica. YT contributed to structure elucidation, NMR analysis, and bioactivities test. JS, SW, and HY were the project leader organizing and guiding the experiments and manuscript writing. All authors contributed to the article and approved the submitted version.

## Conflict of Interest

The authors declare that the research was conducted in the absence of any commercial or financial relationships that could be construed as a potential conflict of interest.

## Footnotes

1 https://blast.ncbi.nlm.nih.gov/Blast.cgi

2 http://itol.embl.de/#

3 http://eztaxon-e.ezbiocloud.net
