# Supplementary material for: Diversity of Cultivable Microbes From Soil of the Fildes Peninsula, Antarctica, and Their Potential Application
Source: Front Microbiol. 2020 Sep 3;11:570836. doi: 10.3389/fmicb.2020.570836 (PMC7495136; doi:10.3389/fmicb.2020.570836)
Supplement: Supplementary file 1 [file Table_1.DOCX]

Supplementary Material

**Diversity of Cultivable Microbes from Soil of the Fildes Peninsula, Antarctica, and Their Potential Application**

***Bailin Cong^1, 3^, Xiaofei Yin^1, 3^, Aifang Deng^1^, Jihong Shen^1^*, Yongqi Tian^2^*, Shaoyun Wang^2^*, Huanghao Yang^2^***

*^1^ The First Institute of Oceanography, State Oceanic Administration, Qingdao 266061, P. R. China.*

*^2^ College of Biological Science and Engineering, Fuzhou University, Fuzhou, 350116, P. R. China.*

*^3^ The two authors contribute equally.*

*** Correspondence:***Jihong Shen (*[*shenjihong@fio.org.cn*](mailto:shenjihong@fio.org.cn)*), Yongqi Tian (*[*tianyongqi@fzu.edu.cn*](mailto:tianyongqi@fzu.edu.cn)*), Shaoyun Wang (*[*shywang@fzu.edu.cn*](mailto:shywang@fzu.edu.cn)*)*

**Table S1.** sampling information

| **Sample number** | **longitude** | **latitude** | **temperature** | **time** |
| --- | --- | --- | --- | --- |
| B1-1  F1-1  K1-1  M1-1  Q2-1 | 58°58.743′W  58°59.443′W  58°58.832′W  58°56.657′W  58°56.020′W | 62°13.797′S  62°11.123′S  62°11.028′S  62°11.542′S  62°12.864′S | -2°C  2°C  4°C  2°C  0°C | 2014-12-17  2014-12-21  2014-12-20  2014-12-21  2014-12-18 |

**Table S2.** ^1^H and ^13^C NMR Data for **1**, **2** (500/125 MHz, respectively in CDCl_3_, *δ* ppm, *J* in Hz).

| **No.** | **1 2** | | | |
| --- | --- | --- | --- | --- |
|  | *δ*_H_ (*J* in Hz) | *δ*_C_ | *δ*_H_ (*J* in Hz) | *δ*_C_ |
| 1 | 7.23, s | 119.1, CH | 7.19, s | 119.0, CH |
| 2 |  | 141.2, C | 7.30, d (9.0) | 141.2, C |
| 3 |  | 152.9, C | 7.15, d (9.0) | 152.4, C |
| 4 |  | 134.7, C |  | 134.1, C |
| 5 |  | 160.8, C |  | 148.7, C |
| 6 | 6.79, d (8.5) | 105.6, CH |  | 149.1, C |
| 7 | 7.58, t (8.5) | 134.7, CH |  | 120.6, CH |
| 8 | 7.01, dd (8.5, 0.5) | 109.9, CH |  | 112.6, CH |
| 9 |  | 152.8, C |  | 153.0, C |
| 10 |  | 120.9, C |  | 120.0, C |
| 11 |  | 112.9, C |  | 117.1, C |
| 12 |  | 157.6, C |  | 150.5, C |
| 13 |  | 179.9, C |  | 179.7, C |
| 14 | 2.41, s | 17.7, CH_3_ | 2.40, s | 17.6, CH_3_ |
| 15 | 4.44, d (7.5) | 72.3, CH_2_ | 4.42, d (7.0) | 72.1, CH_2_ |
| 16 | 5.59, tt (7.0, 1.0) | 120.1, CH | 5.59, tt (7.0, 1.0) | 119.9, CH |
| 17 |  | 138.9, C |  | 138.8, C |
| 18 | 1.78, s | 26.0, CH_3_ | 1.77, s | 25.9, CH_3_ |
| 19 | 1.70, s | 18.3, CH_3_ | 1.69, s | 18.1, CH_3_ |
| 20 | 5.02, s | 57.3, CH_2_ | 5.00, s | 57.2, CH_2_ |
| 21 | 4.02, s | 56.8, CH_3_ | 3.97, s | 61.7, CH_3_ |
| 22 |  |  | 3.89, s | 57.4, CH_3_ |

**Table S3.** ^1^H and ^13^C NMR Data for **3** (500/125 MHz, respectively in CDCl_3_, *δ* ppm, *J* in Hz).

| **No.** | *δ*_H_ (*J* in Hz) | *δ*_C_ |
| --- | --- | --- |
| 1 |  | 164.8, C |
| 2 |  |  |
| 3 | 4.38, t (6.0) | 65.9, CH_2_ |
| 4 | 2.38, t (6.0) | 29.2, CH_2_ |
| 5 |  | 157.8, C |
| 6 | 5.83, q (1.5) | 116.8, CH |
| 7 | 2.01, s | 22.6, CH_3_ |

**Table S4.** ^1^H and ^13^C NMR Data for **4** (500/125 MHz, respectively in CDCl_3_, *δ* ppm, *J* in Hz).

| **No.** | *δ*_H_ (*J* in Hz) | *δ*_C_ |
| --- | --- | --- |
| 1 |  | 157.1, C |
| 2 | 7.57, s | 118.9, CH |
| 3 |  | 129.7, C |
| 4 | 7.59, d (9.0) | 124.7, CH |
| 5 | 7.14, d (9.0) | 121.3, CH |
| 6 |  | 137.1, C |
| 7 |  | 75.3, C |
| 8 | 1.50~1.8, m | 36.8, CH_2_ |
| 9 | 1.51~1.8, m | 16.6, CH_2_ |
| 10 | 1.51~1.8, m  2.45, dt (14.0, 3.0 ) | 33.8, CH_2_ |
| 11 |  | 77.7, C |
| 12 | 1.28, s | 31.3, CH3 |
| 13 | 0.95, s | 24.8, CH3 |
| 14 |  | 172.0, C |
| 15 | 1.50, s | 31.9, CH_3_ |
| OH-6 | 9.37, s |  |
| OH-14 | 10.2, brs |  |


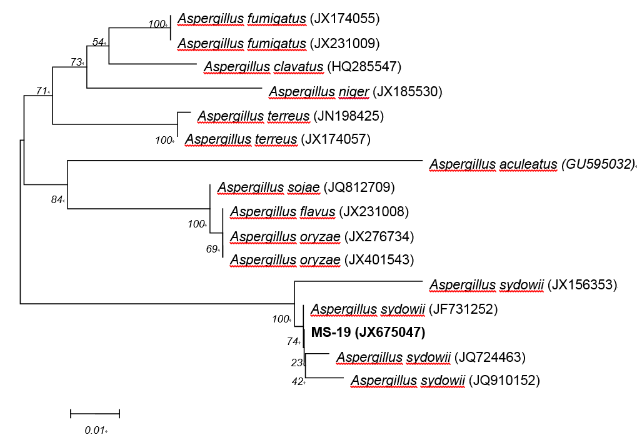

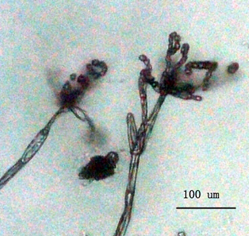

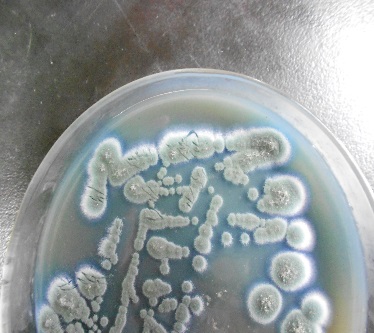


a

b

**Figure S1**. Phylogeny and growth profile of *Aspergillus sydowii* MS-19. **a**. Neighbour-joining tree showing the relationship between ITS sequences from MS-19 and its 15 closest relatives. Bootstrap values for the neighbour-joining analysis with 1000 replicates are shown on the branches. The scale bar represents 0.01 substitutions per amino acid site. **b.** The morphological and microscopic characteristics of Aspergillus sydowii MS-19.


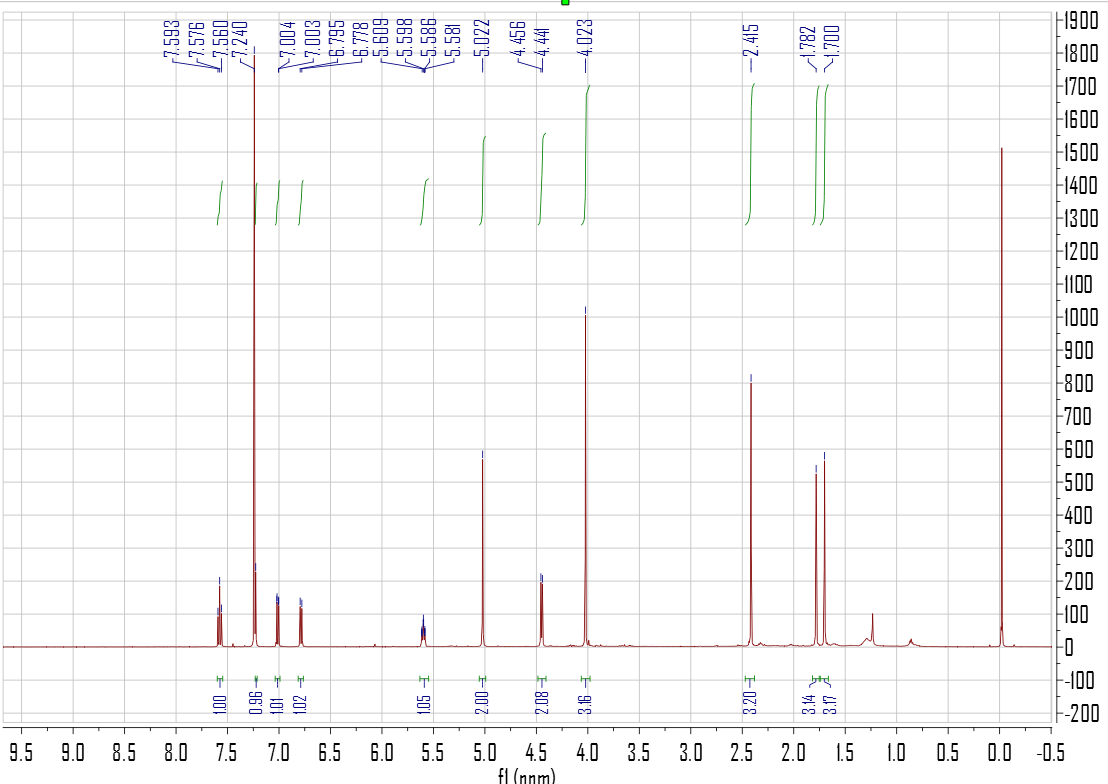


**Figure S2.** The ^1^H NMR spectrum of **1** in CDCl_3_


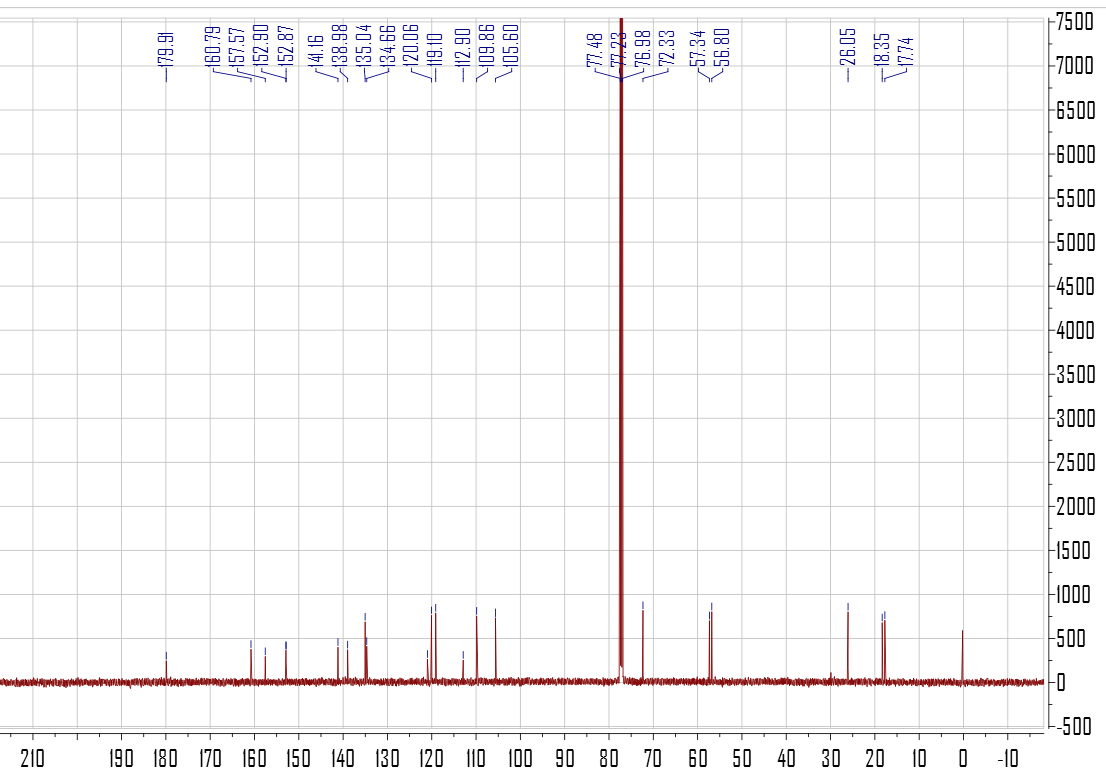


**Figure S3.** The ^13^C NMR spectrum of **1** in CDCl_3_


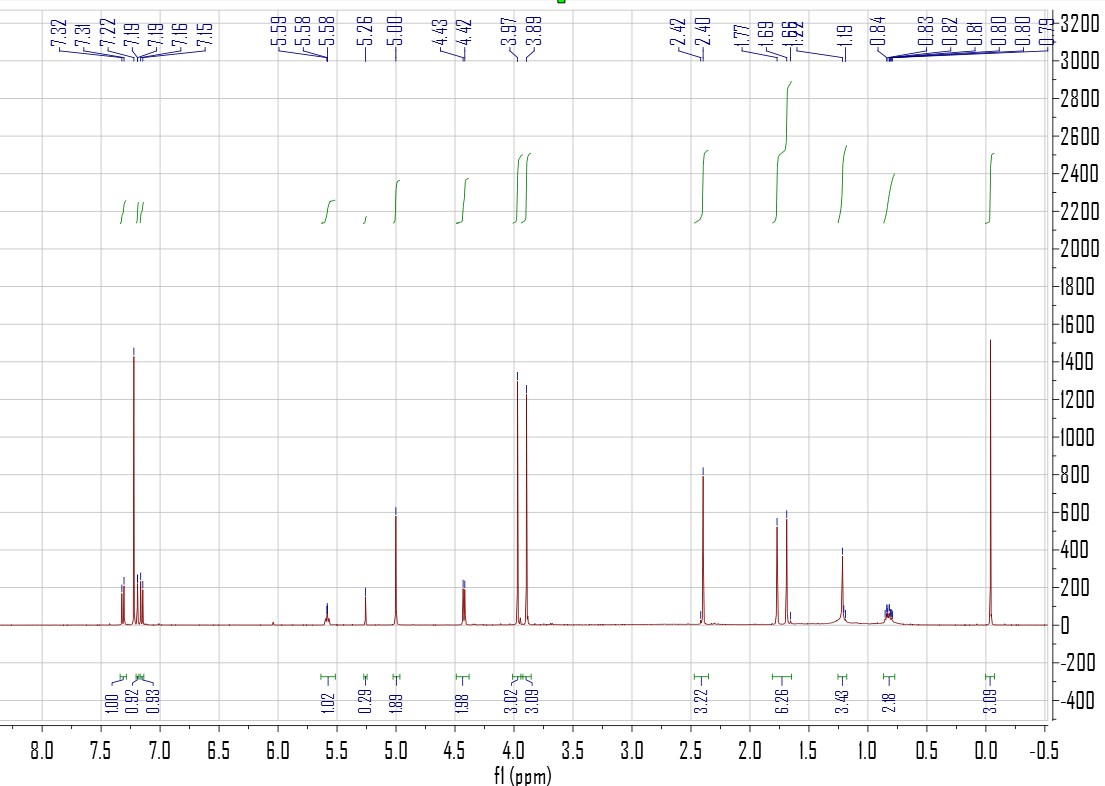


**Figure S4.** The ^1^H NMR spectrum of **2** in CDCl_3_


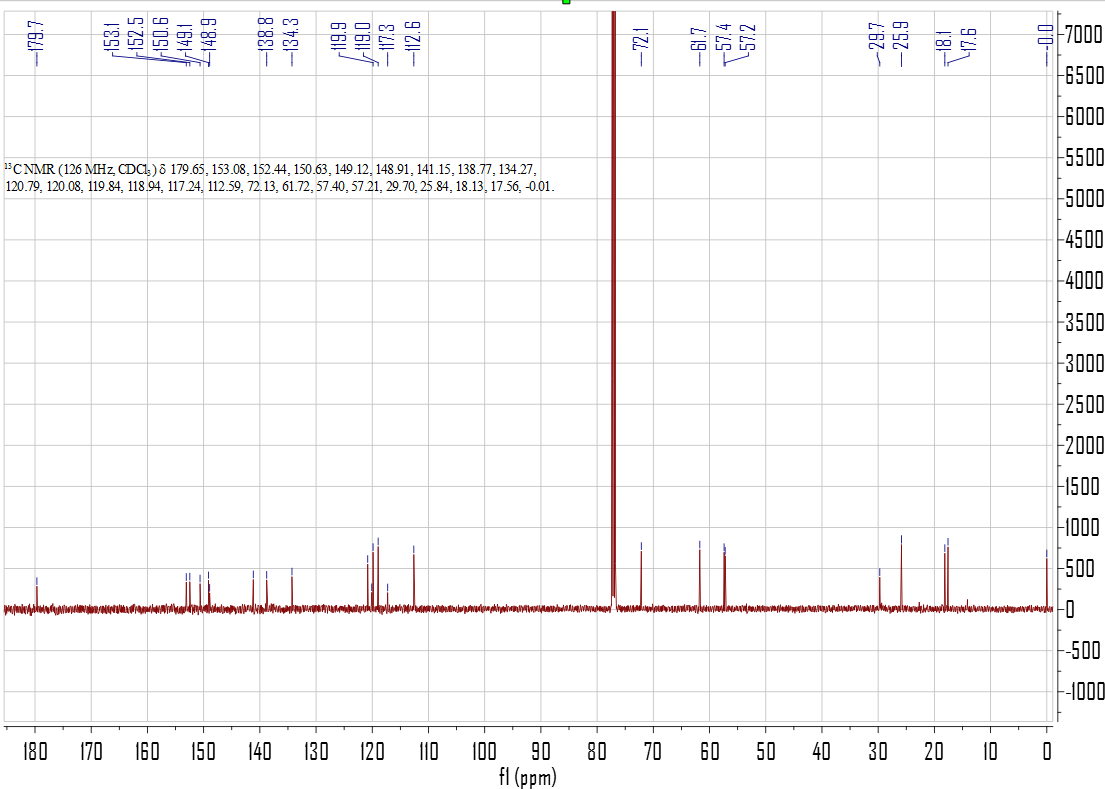


**Figure S5.** The ^13^C NMR spectrum of **2** in CDCl_3_


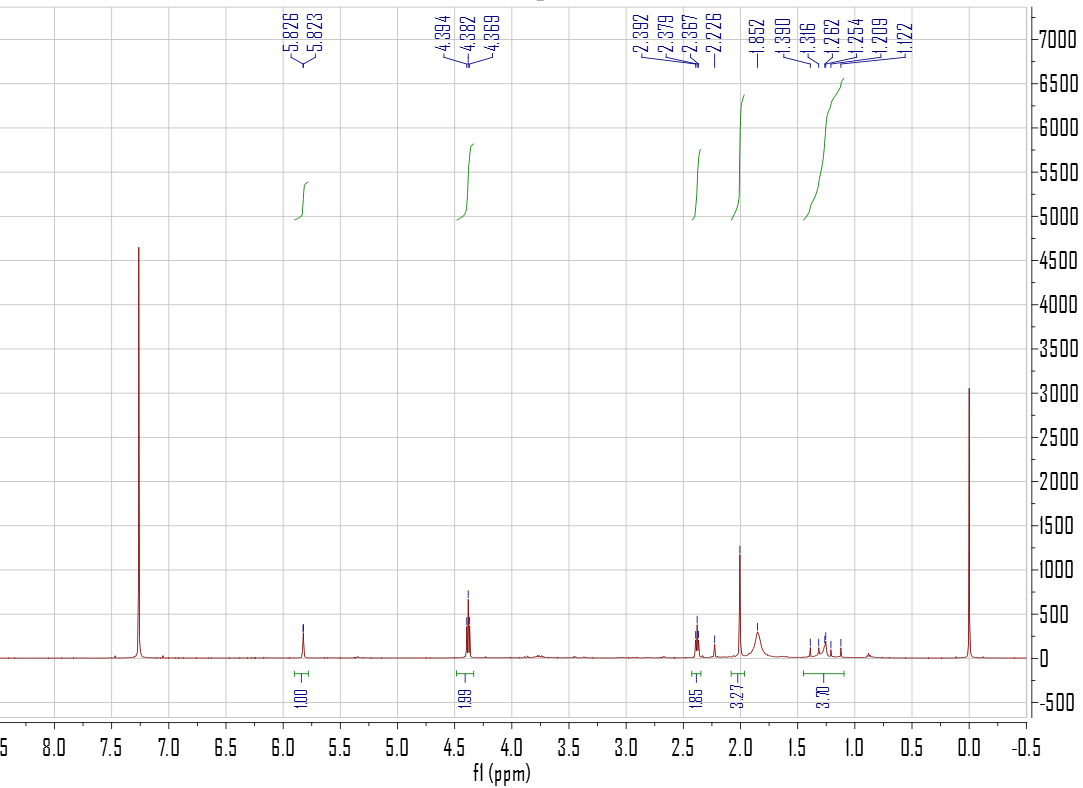


**Figure S6.** The ^1^H NMR spectrum of **3** in CDCl_3_


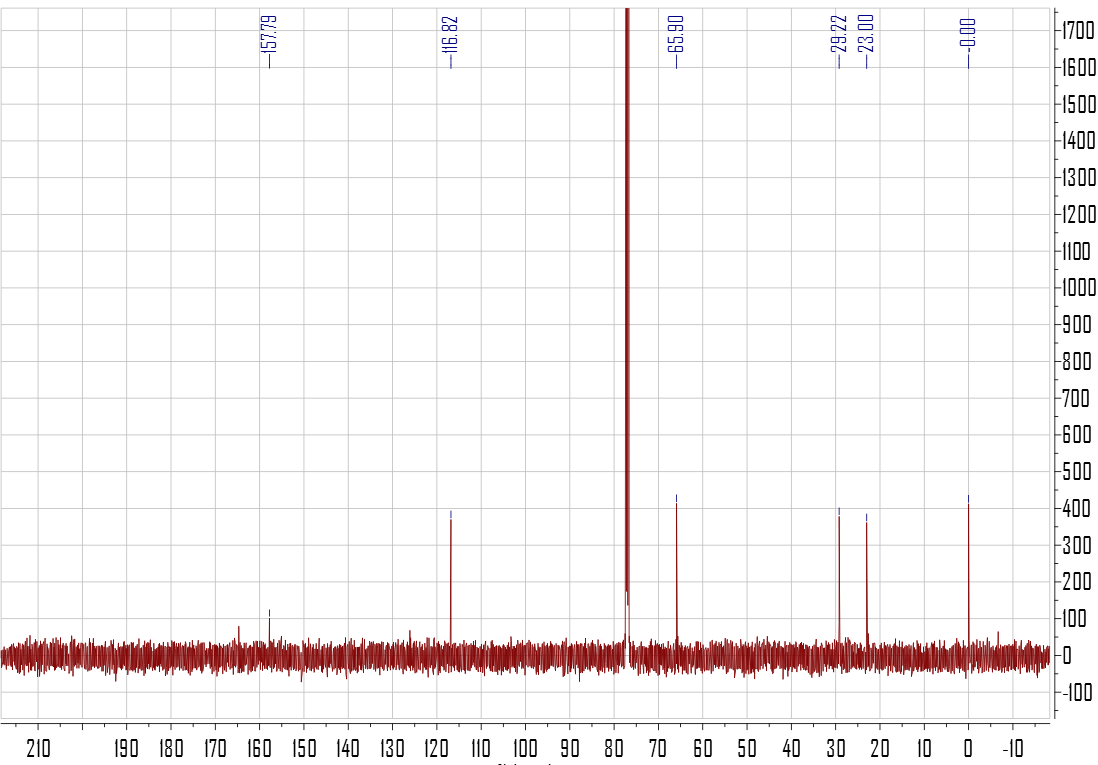


**Figure S7.** The ^13^C NMR spectrum of **3** in CDCl_3_


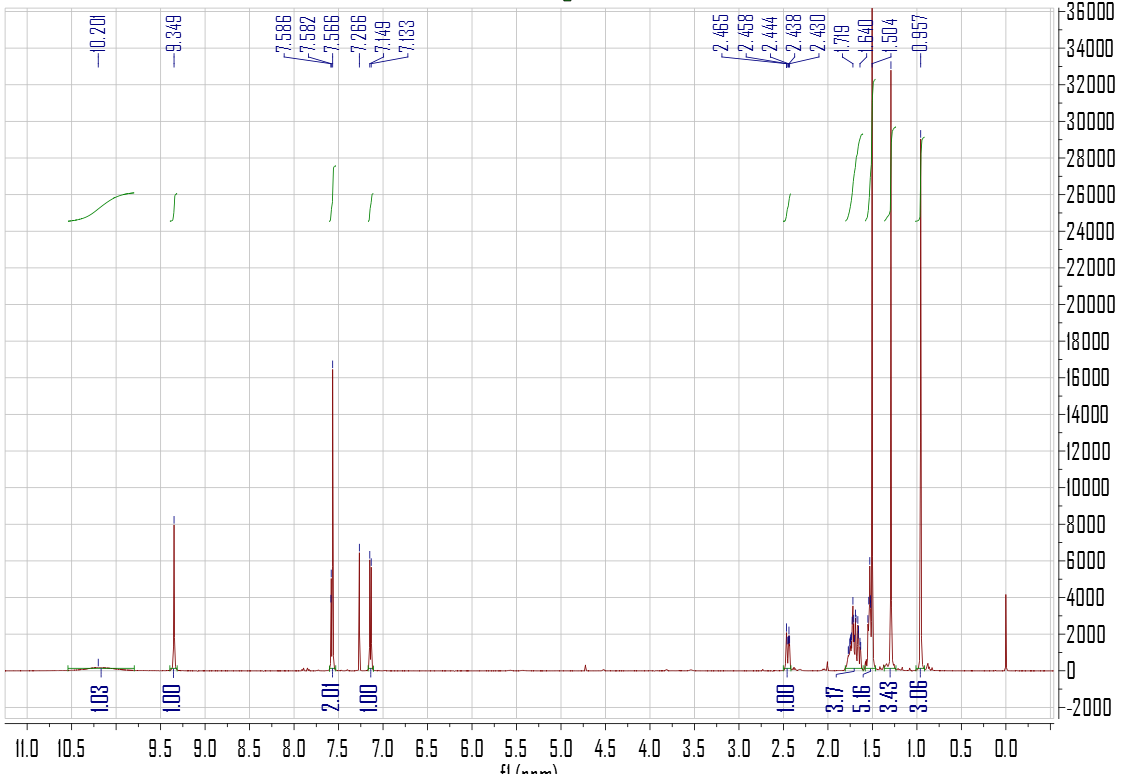


**Figure S8.** The ^1^H NMR spectrum of **4** in CDCl_3_


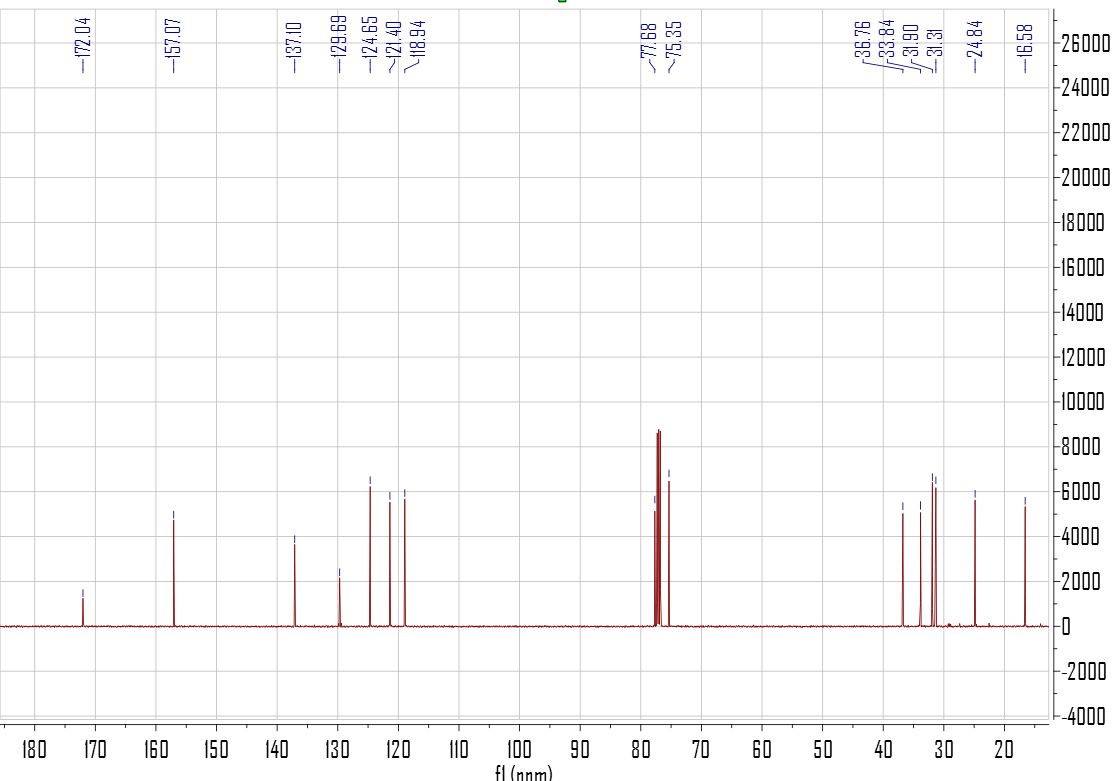


**Figure S9.** The ^13^C NMR spectrum of **4** in CDCl_3_
